# Exploring Genomic Variability in the Mediterranean Buffalo Breed: A Step Towards Custom SNP Array

**DOI:** 10.3390/ani16060922

**Published:** 2026-03-15

**Authors:** Chiara Arcuri, Federica Gabbianelli, Francesca Bencivenga, Gabriella Porcai, Daniele Pietrucci, Ludovica Picarone, Giovanni Vignali, Elvira Toscano, Federica Di Maggio, Leandra Sepe, Marcella Nunziato, Arianna Manunza, Barbara Lazzari, Paolo Cozzi, Francesca Rizzo, Alessandro Weisz, Marharyta Smal, Stefano Biffani, Bianca Castiglioni, Giovanni Paolella, Francesco Salvatore, Alessandro Rullo, Salvatore Rubinacci, Gianfranco Cosenza, Mayra Gómez Carpio, Roberta Cimmino, Gabriele Di Vuolo, Leopoldo Iannuzzi, Marco Milanesi, Giovanni Chillemi

**Affiliations:** 1 Department for Innovation in Biological, Agro-Food and Forest Systems—DIBAF, University of Tuscia, 01100 Viterbo, Italy; chiara.arcuri@unitus.it (C.A.); federica.gabbianelli@unitus.it (F.G.); francesca.bencivenga@unitus.it (F.B.); porcai@unitus.it (G.P.); daniele.pietrucci@unitus.it (D.P.); ludovica.picarone@unitus.it (L.P.); giovanni.vignali@unitus.it (G.V.); chillemi@med.uniroma2.it (G.C.); 2 Ceinge Biotecnologie Avanzate-Franco Salvatore, Via Gaetano Salvatore, 486, 80145 Napoli, Italy; toscano@ceinge.unina.it (E.T.); dimaggio@ceinge.unina.it (F.D.M.); leandra.sepe@unina.it (L.S.); nunziato@ceinge.unina.it (M.N.); giovanni.paolella@unina.it (G.P.); salvator@unina.it (F.S.); 3 Department of Molecular Medicine and Medical Biotechnologies, University Federico II, Via Sergio Pansini, 5, 80131 Napoli, Italy; 4 Institute of Agricultural Biology and Biotechnology, National Research Council, Via Alfonso Corti 12, 20133 Milano, Italy; arianna.manunza@ibba.cnr.it (A.M.); barbara.lazzari@ibba.cnr.it (B.L.); paolo.cozzi@ibba.cnr.it (P.C.); biffani@ibba.cnr.it (S.B.); bianca.castiglioni@ibba.cnr.it (B.C.); 5 Department of Animal Science, Food and Nutrition, Faculty of Agricultural, Food and Environmental Sciences (DiANA), Università Cattolica del Sacro Cuore, Via E. Parmense 84, 29122 Piacenza, Italy; 6 Laboratory of Molecular Medicine and Genomics, Department of Medicine, Surgery and Dentistry ’Scuola Medica Salernitana’, University of Salerno, via S. Allende snc, 84081 Baronissi, Italy; frizzo@unisa.it (F.R.); aweisz@unisa.it (A.W.); masmal@unisa.it (M.S.); 7 Dipartimento di Scienze Medico-Veterinarie, Università Degli Studi di Parma, 43100 Parma, Italy; 8 Neatec S.p.A., Via Campi Flegrei, 34, 80078 Pozzuoli, Italy; a.rullo@neatec.it (A.R.); s.rubinacci@neatec.it (S.R.); 9 Department of Agricultural Sciences, University of Naples Federico II, Piazza Carlo di Borbone, 1, 80055 Portici, Italy; gianfranco.cosenza@unina.it; 10 Associazione Nazionale Allevatori Specie Bufalina—ANASB, Via Petrarca, 42-44, 81100 Caserta, Italy; m.gomezcarpio@anasb.it (M.G.C.); r.cimmino@anasb.it (R.C.); direzione@anasb.it (G.D.V.); 11 Institute of Animal Production System in Mediterranean Environment (ISPAAM), National Research Council (CNR), 80055 Naples, Italy; leopiannuzzi949@gmail.com; 12 Department of Experimental Medicine, University of Rome “Tor Vergata”, 00133 Rome, Italy

**Keywords:** Mediterranean buffalo, WGS, biallelic SNP dataset, SNP chip, river buffalo, swamp buffalo, long-reads, ONT

## Abstract

Genomic improvement and sustainable breeding of Mediterranean buffalo are hampered by the lack of breed-specific genomic resources compared with bovine. To address this gap, we aimed to identify a comprehensive set of high-confidence single-nucleotide polymorphisms (SNPs) in Mediterranean buffalo and evaluate their informativeness across other buffalo populations. A total of 58 whole-genome sequencing samples, from three different sets, were merged through the integration of short- and long-read sequencing technologies. Variants, both unique and shared among datasets, were identified using a combination of bioinformatic tools to increase the reliability of the dataset. From these, over 11 million high-confidence biallelic SNPs were identified in the Mediterranean breed. The same SNPs were also tested in additional populations (other rivers and swamps) to evaluate their polymorphism and missingness. These results provide a robust genomic resource for Mediterranean buffalo, overcoming the limitations of bovine-derived genotyping tools available today. The identified SNPs dataset lays the basis for the development of a breed-specific SNP array, providing a dense and informative set of markers that could support cost-effective SNP chip development compared with existing arrays and sequencing technologies. This resource will facilitate more accurate genomic selection, precision breeding, and the conservation of genetic diversity in the Mediterranean buffalo population.

## Simple Summary

Mediterranean buffalo is an important breed for the Italian livestock sector and agri-food system. However, genetic resources for this breed, and for buffalo in general, are limited compared with bovines. In this study, we analyzed the DNA of Mediterranean buffaloes to identify small differences in the genetic code, called single-nucleotide polymorphisms (SNPs), which can help distinguish individuals and associate them with phenotypes of interest for breeding. By combining different sequencing technologies and methodologies, we detected over eleven million high-quality variants in the Mediterranean breed. We also evaluated these variants in other buffalo populations (other rivers and swamps) to obtain a database for the buffalo species. Our results show that these markers cover the entire genome uniformly (with approximately 1 marker every 180 bp) and may reliably reflect buffalo genetic diversity. These results are instrumental in the development of buffalo genomic technologies, sustainable breeding, improving animal health and productivity, and contributing to the conservation of Mediterranean buffalo diversity.

## 1. Introduction

The water buffalo (*Bubalus bubalis*) is a vital livestock species primarily found in eastern and tropical regions where it is largely bred for both milk and meat production [1,2]. It comprises several species and subspecies, some even with a different diploid chromosome number. Two of them are the most important from an economic point of view, largely widespread: the river buffalo (2n = 50) and the swamp buffalo (2n = 48) [3,4].

At the global scale, the water buffalo population has undergone a marked expansion over recent decades. Early estimates from the 1980s reported approximately 150 million animals [5], while more recent assessments indicate a worldwide population of ~206–208 million animals, of which 95–96% belong to the river type [3].

Updated Food and Agriculture Organization (FAO) evaluations confirm that river buffalo numbers continue to grow, whereas swamp populations have shown a declining trend over the last decade [6]. In this context, although the number of buffalo in Italy represents a relatively small share of the national livestock population [7], the Italian Mediterranean buffalo nonetheless retains a significant economic role, with a production reaching a value of €529 million in 2023 (a 15% increase over 2021 [8]). Buffalo milk production is, in fact, second only to bovine milk [9]. According to recent data from the FAO and Italian National Institute of Statistics (ISTAT) (2024), this breed plays a central role in Italy’s dairy sector (particularly in the Campania region), with production exceeding 2.4 million tons in 2024. Italy leads the European market for PDO dairy products, such as “Mozzarella di Bufala Campana” and “Ricotta di Bufala Campana” [10,11].

Traditionally bred in Italy since the Middle Ages, the breed has expanded steadily and now accounts for about 432,000 animals across 2485 farms, mainly in southern regions [12,13]. Nowadays, Mediterranean buffalo milk and whey are valued for the remarkable nutritional qualities of these foods, as well as their potential for future applications in the food and nutraceutical sectors [14]. This trend is further reinforced by the rising demand for healthier food options. There is a clear need to establish strategies that encourage the consumption of functional dairy products, especially those with probiotic properties, aligning with current consumer health trends [15]. This evolution in consumer preferences is significantly influencing breeding goals: selection strategies are no longer focused solely on maximizing output, but also on improving production efficiency and product quality [16].

To meet the evolving needs of the sector, genomic selection is a powerful tool, enabling breeders to identify and propagate animals with favorable genetic profiles for production traits and functional properties [17]. A fundamental genomic tool that has become particularly widespread and has revolutionized genetic research in livestock and also in *B. bubalis* is the SNP chip (single-nucleotide polymorphism array) technology. This enables economically important applications such as trait analysis [18,19], genomic value prediction [20], genetic diversity assessment [21], and identification of selection signatures [22]. Since genomic evaluation has already been implemented in the official breeding programs of the Italian Mediterranean buffalo, the need for accurate and species-specific genomic tools is even more critical [23].

The medium-density SNP array currently available (Axiom^®^ Buffalo Genotyping Array 90K) was developed in 2017 using data from both river and swamp buffalo, but was aligned to the *Bos taurus* reference genome, as a buffalo reference genome was not available at the time [24]. This array has several limitations because it (a) was designed mainly for genetic diversity studies, (b) does not have a uniform distribution of the marker in the bufaline genome due to the non-use of a species-specific reference, (c) was not designed to be optimized for the Mediterranean Genomic Selection program, and (d) does not include markers from the Y chromosome.

To overcome these limitations, one of the aims of this study is to establish the basis for the development of a next-generation SNP chip, specifically tailored to the Italian Mediterranean buffalo and genomic selection use, identifying markers suitable for this purpose. To create a new array or improve an existing one, new variants, in particular, biallelic SNPs, need to be included. The SNPs could be identified in already existing variant databases or “discovered” using sequencing data.

In this project, sequence data were analyzed with the objective of building a new variant dataset specific to Mediterranean water buffalo, but, at the same time, with information on other buffalo types, providing a valuable resource to assess the genetic variability of the breed and support genomic selection studies.

## 2. Materials and Methods

**Figure 1:**
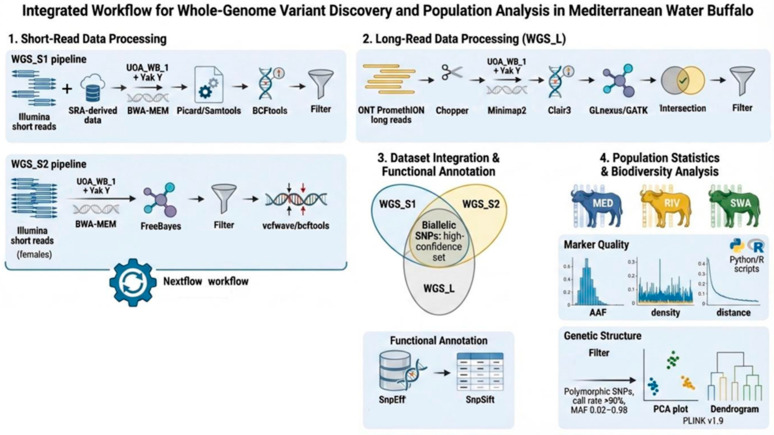
Schematic workflow of the applied pipeline. The image was made using the FigureLabs.ai.

To identify small genetic variants, including single-nucleotide polymorphisms (SNPs), insertions/deletions (InDels), and multi-nucleotide polymorphisms (MNPs), specific to the Mediterranean water buffalo, three independent whole-genome sequencing (WGS) datasets were analyzed. The Mediterranean buffalo blood samples used in this study were from animals registered in the herdbook of the Italian Mediterranean buffalo breed (ANASB). Two variant datasets were based on Illumina short-read sequencing, while the third one was based on Oxford Nanopore Technologies (ONT) long-read sequencing, produced elsewhere by the F.S. and G.Pa. groups. The analyzed individuals included not only Mediterranean buffaloes but also animals from river (Murrah, Jaffrabadi, and Nili-Ravi) [25] and swamp breeds [26]. In the following paragraphs, we specify the details and the bioinformatic protocols used for each (Figure 1).

### 2.1. Whole-Genome Sequencing Datasets

#### 2.1.1. WGS_S1 Short-Read Data

Two male DNA pools were prepared for the identification of Y-chromosome variants. The pool strategy was used to maximize the variability representativeness inside the MED population, reducing the sequencing cost. However, these data could not be used to evaluate single-individual performances. The first pool (WGS_S1_P1) was generated by combining genomic DNA from 18 individuals, which was then sonicated and processed as a single library. The second pool (WGS_S1_P2) consisted of three samples, obtained by extracting genomic DNA from individual blood samples of bulls currently approved for artificial insemination by Associazione Nazionale Allevatori Specie Bufalina (ANASB), using the DNeasy^®^ Blood & Tissue Kit (Qiagen^®^, Hilden, Germany), according to the manufacturer’s protocol. These samples were processed separately during library preparation and subsequently pooled. Sequencing libraries were constructed using the NEBNext^®^ Multiplex Oligos for Illumina^®^ (96 Unique Dual Index Primer Pairs) (New England Biolabs, Ipswich, MA, USA), following the manufacturer’s instructions. Sequencing was performed on an Illumina platform in paired-end mode (2 × 150 bp), yielding an average total genome coverage, the percentage of covered bases, of approximately 30× for both pools.

Whole-genome short-read data from 22 male buffaloes were retrieved from the NCBI Sequence Read Archive and ENA (European Nucleotide Archive). This dataset included 11 Mediterranean individuals, 5 animals from other river-type groups (2 Murrah, 1 Jaffrabadi, 1 Nili-Ravi and 1 India) and 6 animals from a swamp-type group (Chinese origin). The sequencing depth ranged from 3× to 40× across samples. A detailed list of accession IDs is provided in Table S1.

Hereafter, this combined dataset of in-house pool-seq and public short-read data will be referred to as WGS_S1.

#### 2.1.2. WGS_S2 Short-Read Data

The IBMI genomic index (Italian Mediterranean Buffalo Index) is the official selection index for the Italian Mediterranean Buffalo breed and is computed by ANASB [27]. This composite index integrates both productive and functional traits, aiming to identify individuals that combine high milk performance with longevity and overall farm efficiency. For this study, a total of 24 females were selected based on their IBMI genomic values, collected during the first semester of 2023. These individuals were chosen because they exhibit desirable productive attributes (milk and mozzarella yield potential) together with favorable functional characteristics, particularly udder conformation and locomotor soundness. To ensure adequate genetic diversity within the subset, the selected animals originated from different farms and genetic lines. The animals were classified into two groups according to their IBMI genomic values: those with an IBMI < 100 were assigned to the low group, while those with an IBMI > 100 were placed in the “high” group, with 100 representing the population reference mean. Genomic DNA was extracted and sequenced using Illumina NovaSeq 6000 (Illumina, Inc, Hayward, CA, USA) (2 × 100 bp), with an average coverage of 30–40×. Sequencing and processing followed the nf-resequencing-mem workflow [28]. Hereafter, this dataset will be referred to as WGS_S2.

#### 2.1.3. WGS_L Long-Read Variant Dataset Data

The long-read variant dataset comprises eight male Mediterranean buffalo individuals, which are unrelated, sequenced using the Oxford Nanopore PromethION24 platform (Oxford Nanopore Technologies, Oxford, UK). Genomic DNA was extracted from blood using an optimized protocol designed to maximize both the quality and yield of high-molecular-weight DNA, which is essential for long-read sequencing applications. WGS libraries were prepared as described in the following references [29,30].

In particular, the dataset was used to validate and support the variants identified through short-read sequencing. Hereafter, this variant dataset will be referred to as WGS_L.

### 2.2. Reference Genome

All read alignments and variant calling steps were performed using a composite reference genome consisting of UOA_WB_1 Mediterranean water buffalo assembly [31] and the Y chromosome from Yak (*Bos grunniens*) [32] due to the lack of the Y chromosome in the water buffalo genome.

The alignment evaluation was performed for all three datasets using the same pipeline. For each sample, coverage was calculated with samtools coverage [33]. Sequencing depth was estimated by multiplying the number of mapped reads, obtained from the samtools idxstats output, by their average read length, derived from the samtools stats report, and dividing the result by the total genome size. The genome size was calculated as the sum of the lengths of all contigs.

### 2.3. Variant Calling Pipeline

The variant calling process differed slightly across the three datasets, reflecting differences in data characteristics. In all cases, the analyses primarily focused on the identification of the biallelic single-nucleotide polymorphisms (SNPs).

#### 2.3.1. WGS_S1 Variant Calling Pipeline

Reads were aligned using the BWA-MEM [34] software v0.7.17. The resulting BAM files were processed with Picard v2.25.7 [35] and Samtools v1.23 for sorting, indexing, duplicate marking, and read group assignment.

Variant calling was performed using BCFtools v1.13 [36]. The mpileup function was used to retain alternative allele depth (AD) and depth per sample (DP). Variants were called using bcftools call in multiallelic mode. The datasets derived from SRA reads and Pool-seq experiments were then treated separately to apply dataset-specific filters using VCFtools v0.1.16 [37]. For the SRA-derived dataset, variants with a minimum read depth (--minDP) of 5 were retained. After filtering, the two datasets were merged, and a global filter for the maximum proportion of missing data (--max-missing) of 0.1 was applied. The choice of the --minDP threshold was informed by a preliminary assessment of alignment quality and sequencing depth, which was generally low across the analyzed samples. Pool-seq samples were excluded for this filtering step due to the high percentage of duplicate reads observed after alignment.

#### 2.3.2. WGS_S2 Variant Calling Pipeline

Reads were aligned to the reference genome using BWA-MEM v0.7.18, and the resulting BAM files were processed with Picard v3.3.0 and Samtools v1.21 to retain only uniquely mapped reads.

Variant calling was carried out using FreeBayes 1.3.8 [38]. SNPs were subsequently filtered using VCFtools with the following parameters: a minimum genotype quality (--minGQ) of 30, a minimum read depth (--minDP) of 10, and a maximum proportion of missing data (--max-missing) set to 0.1. The --minDP and --minGQ thresholds were chosen based on a preliminary evaluation of alignment quality and sequencing depth, which was generally high across the analyzed samples. Variants identified by Freebayes as multi-nucleotide polymorphisms (MNPs) were transformed into SNPs with a step of normalization by vcfwave v1.0.13 and bcftools norm and vt decompose_blocksub v0.57721, in order to enable direct comparison with other datasets containing only biallelic SNPs. All these steps were implemented and managed through the Nextflow [39] pipeline https://github.com/cnr-ibba/nf-resequencing-mem (accessed on 8 March 2026), ensuring reproducibility and scalability of the workflow [40].

#### 2.3.3. WGS_L Variant Calling Pipeline

Initial filtering of ONT reads was performed using Chopper v0.12.0b [41], excluding reads with a quality score of less than 10 or a length of less than 150 bp. High-quality reads were aligned with Minimap2 v2.30 [42]. BAM files were sorted and indexed with Samtools, and quality was assessed using NanoPlot v1.19.0 [41].

Variant calling was performed using Clair3 v0.2.2 [43] in GVCF mode. Joint genotyping per chromosome was conducted using Glnexus v1.4.1 [44] and GATK v4.6.2.0 [45]. Variants common to both tools were retained using BCFtools.

The filtering criteria included biallelic SNPs and a genotype quality (--minGQ) of more than 20, a depth (--minDP) of more than 5, and presence in at least 2 out of 8 individuals, using VCFtools software. Also, here, the --minDP and --minGQ thresholds were chosen based on a preliminary evaluation of alignment quality and sequencing depth.

### 2.4. Variant Dataset Merging and Variant Annotation

The three datasets (WGS_S1, WGS_S2, and WGS_L) were compared using BCFtools isec to identify variants unique to each dataset as well as those shared among all three. Unique variants were analyzed by type and filtered for biallelic SNPs, while variants common to all datasets, also filtered for biallelic SNPs, were used to build a high-confidence biallelic SNP dataset.

Common variants were annotated using the SnpEff pipeline [46]. A custom SnpEff database was constructed based on the UOA_WB_1 genome assembly. Variant effect summaries were generated using SnpSift [46] and complemented with additional post-processing scripts.

### 2.5. Statistics on Biallelic SNP Dataset

To evaluate marker quality across genetic groups, the VCF files were subdivided into three breed-based subsets: Mediterranean (MED—47 animals), rivers, excluding MED (RIV—5 animals), and swamp (SWA—6 animals). Genotype missingness and alternative allele frequency (AAF) were calculated using custom Python v3.12.10 and R v4.5.2 scripts. Specifically, AAF was computed for each genetic group as the ratio between the number of reads supporting the AD and DP for that variant, as reported in the VCF file, for all the individuals/pools in the group. Further statistical analyses were conducted to evaluate key characteristics of the variants, including marker density and average inter-marker distance across the genome, using custom Python and R scripts. These metrics provided an overview of SNP coverage and quality, informing downstream analyses.

### 2.6. Statistics on Biodiversity

To assess genetic diversity and population structure, we excluded samples with high missingness (greater than 0.5). Only highly reliable and polymorphic SNPs in the Mediterranean group were retained by filtering for variants in Mediterranean samples with a call rate greater than 90% and an allele frequency between 0.02 and 0.98. Principal component analysis (PCA) and hierarchical clustering dendrogram were performed using the PLINK v1.9 software [47].

## 3. Results

### 3.1. Alignment Evaluation

Reads from the WGS_S1, WGS_S2, and WGS_L datasets were successfully aligned to the reference genome, with consistently high mapping rates (99.35% for WGS_S1, >99.25% for WGS_S2, and 96.44% for WGS_L; Table S2). The mean genome coverage exceeded 96% across all datasets. For WGS_S1, the mean genome coverage was 96.38%, with sequencing depths ranging from 3× to 40×. The WGS_S2 dataset showed a higher mean genome coverage (98.91%) and sequencing depths between 28× and 56×. The WGS_L dataset exhibited a genome coverage of 97.02%, with sequencing depths ranging from 25× to 63×. Duplicate reads were generally detected at low levels across samples, although higher duplication rates were observed in the pooled WGS_S1 samples (Table S2).

### 3.2. Variant Discovery in the Complete Raw Dataset

Variant calling identified approximately 37, 39, and 15 million variants in WGS_S1, WGS_S2, and WGS_L, respectively, for a total of ~41 million unique variants, of which ~14 million were shared among all three datasets. Across datasets, biallelic SNPs represented the vast majority of variants (92.43%), followed by InDels (6.80%), while multi-allelic variants were rare (0.09%; Figure S1).

**Figure 2:**
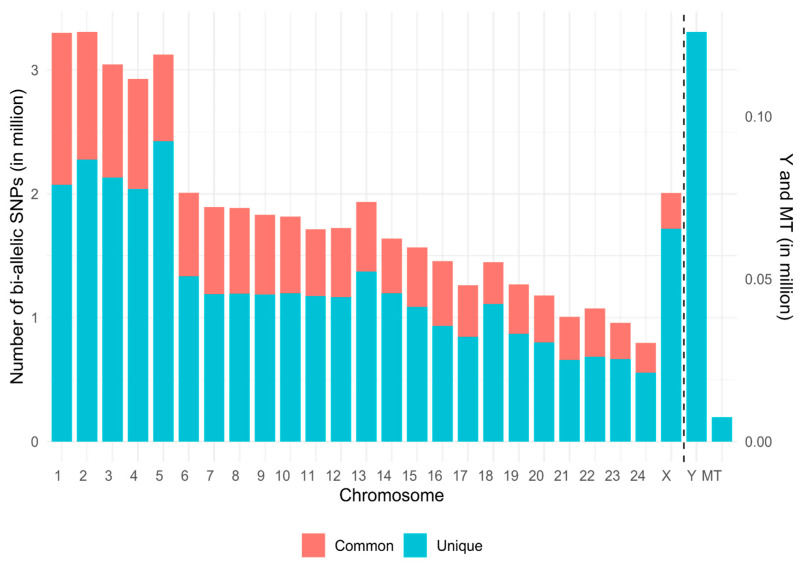
Distribution of unique and common biallelic SNPs across river buffalo chromosomes. The stacked bar plot shows the number of identified variants per chromosome, classified as unique (blue) or common (shared across the three datasets; orange). Chromosomes are arranged sequentially from 1 to 24, including the X, Y (from *B. grunniens*), and mitochondrial (MT) chromosomes. The dashed line separates Y and MT, which are displayed on a different scale to better appreciate their number of variants. Note that they have only unique variants because variants on chromosome Y were identified only in the WGS_S1 dataset (in WGS_S2, all samples are female), while MT is not represented in the WGS_L dataset due to the inability of the sequencing technology to accurately map to very short sequences.

To generate a next-generation SNP chip, only biallelic SNPs with high polymorphism and low missingness were retained for further analyses. Among these, more than 38 million unique and 14 million common variants were identified across the three WGS datasets (Figure S2). The chromosomal distribution of both unique and common biallelic SNPs across the reference genome is reported in Figure 2. The final dataset consisted of biallelic SNPs common to all three datasets for autosomal and X chromosomes, those from WGS_S1 for the Y chromosome, and those from WGS_S1 and WGS_S2 for the mitochondrial genome.

### 3.3. High-Confidence Biallelic SNP Dataset

At the chromosomal level, notable differences in marker density were observed. Chromosome 22 exhibited the average highest SNP density (6282 markers/Mbp) and, as a consequence, the shortest average inter-marker distance (159 bp). In contrast, chromosome 18 showed the average lowest density (5108 markers/Mbp) and, as a consequence, the largest average distance (196 bp) (Table S3).

**Figure 3:**
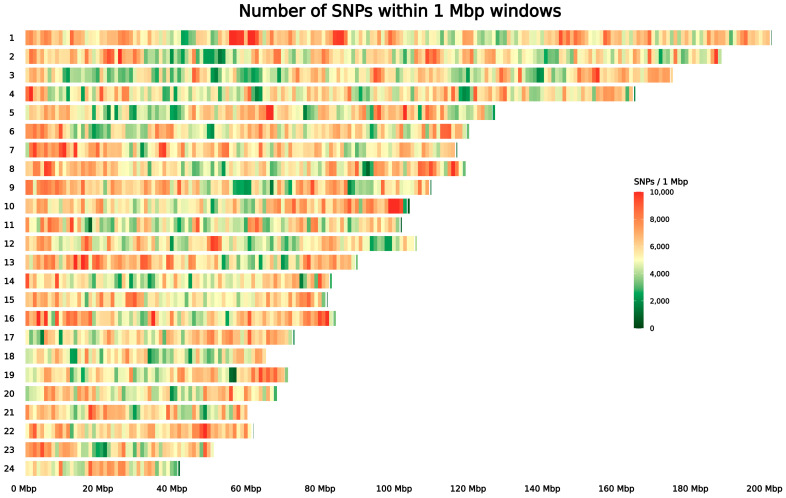
Heatmap showing the genome-wide distribution of biallelic SNPs across the 24 autosomes, calculated in non-overlapping 1 Mbp windows. Each tile represents a genomic window, and color intensity reflects the number of SNPs within that region. Warmer colors (yellow–red) indicate regions with higher marker density, whereas cooler colors (green–dark green) correspond to SNP-poor regions. The extreme color intensities highlight genomic segments characterized by particularly high or low variant density.

Across the autosomal genome, the mean SNP density was estimated at approximately 5624 SNPs per 1 Mbp (±1680 SD) (Figure 3). Among the autosomes, the most SNP-dense regions were located on chromosomes 16 (35.00–36.00 Mbp; N = 12,421), 13 (14.00–15.00 Mbp; N = 11,980), and 5 (66.00–67.00 Mbp; N = 11,773), whereas the SNP-poorest autosomal windows were located on chromosomes 19 (57.00–58.00 Mbp; N = 737), 11 (18.00–19.00 Mbp; N = 776), and 8 (93.00–94.00 Mbp; N = 941).

**Figure 4:**
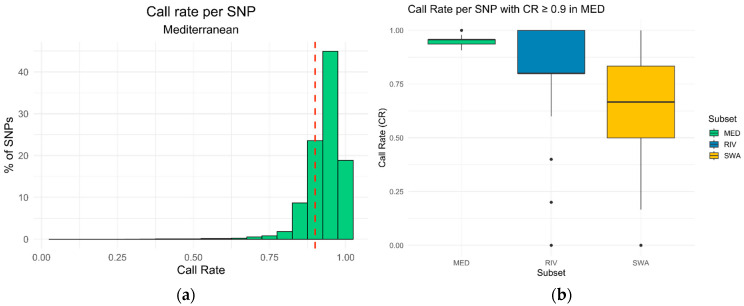
Call rate distribution per SNP in Mediterranean and breed subsets. (**a**) Distribution of the call rate (CR) per SNP in the Mediterranean (MED) subset. The histogram shows the percentage of SNPs across different CR values. The dashed vertical line indicates the CR threshold of 0.9, used to select high-quality SNPs. Most SNPs exhibit high reliability, with over 78% showing CR ≥ 0.9. (**b**) Distribution of the call rate (CR) per SNPs across the different breed subsets using only the SNPs with CR ≥ 0.9 in the MED group. Overall, the boxplots indicate consistent CR values among groups, with higher variability observed in the SWA subset and only a limited number of outlier SNPs showing lower reliability.

To assess marker performance across genetic groups, samples were grouped into Mediterranean (MED; n = 47), river (RIV; n = 5), and swamp (SWA; n = 6) subsets. For each subset, the call rate (CR) was calculated (Figure 4a). A CR threshold of 90% was applied to identify highly reliable SNPs in the MED group, and over 78% of the SNPs exhibited a CR greater than the threshold. The performance of these markers was then compared across the other breed subsets (Figure 4b). Overall, the boxplots indicated interquartile CR values (Q1–Q3) ranging from approximately 80% to 100% in the river population and from 50% to 83% in the swamp population, with only a limited number of outlier SNPs exhibiting lower CR values. Specifically, the percentage of these biallelic SNPs in the RIV group with a CR above 90% was 36.69%, compared with just 9.28% in the SWA group.

**Figure 5:**
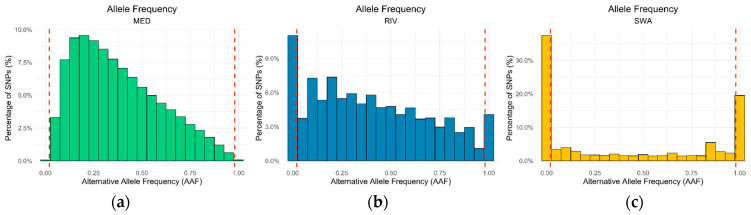
Distribution of alternative allele frequencies across Mediterranean, river, and swamp buffalo populations. Frequency distribution of alternative allele frequencies (AAFs) for SNPs within (**a**) the Mediterranean population; (**b**) the river population; and (**c**) the swamp population. Dashed red vertical lines indicate the lower and upper AAF thresholds applied to select polymorphic markers.

To assess the distribution of alternative alleles, the alternative allele frequency (AAF) was analyzed. In the MED population (Figure 5a), approximately 99.9% of common SNPs had AAF values between 0.02 and 0.98; these SNPs were considered polymorphic and constituted the set of highly polymorphic markers selected for further analysis. This set of polymorphic SNPs was then tested in the other genetic group: in the RIV population, 85.1% of these markers were polymorphic, as defined before (Figure 5b), while in the SWA population, only 43.8% fell within this range (Figure 5c).

After quality control procedures, the final working dataset comprised 53 animals (42 MED; 5 RIV; and 6 SWA) and 11,249,692 high-quality, polymorphic SNPs, which were then used for subsequent analyses.

**Figure 6:**
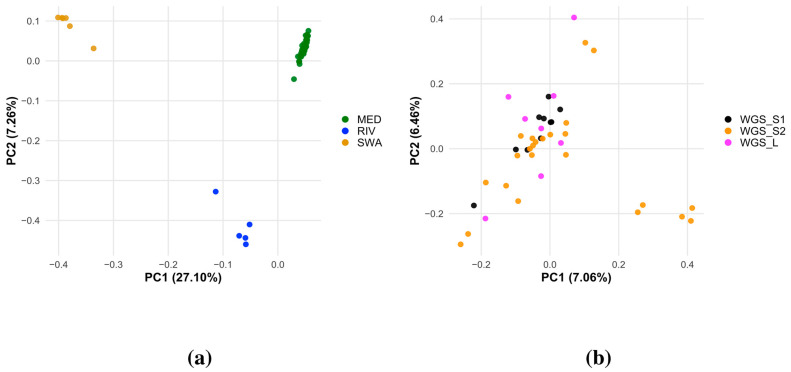
(**a**) Principal component analysis (PCA) based on SNP genotypes (PC1 vs. PC2). Each point represents an individual: green indicates the Mediterranean population, blue the other river populations, and yellow the swamp population. The relative distances between points reflect the genetic similarity or divergence among individuals. (**b**) Principal component analysis (PCA) based on SNP genotypes (PC1 vs. PC2) for only Mediterranean samples. Each point represents an individual (43 samples), excluding only one sample with high missingness. The three sequencing sets are color-coded: WGS_S1 in black, WGS_S2 in orange, and WGS_L in magenta.

Population structure and genetic differentiation were evaluated using PCA (Figure 6) and hierarchical clustering (Figure S3). In the first PCA, which included all breeds (Figure 6a), the first two principal components explained 27.10% and 7.26% of the total variance. Distinct clustering of individuals corresponding to the MED, RIV, and SWA populations was observed. In contrast, the third principal component (PC3), explaining 5.08% of the total variance, highlighted subtle differentiation within the MED group (Figure S4). Analysis of the PCA computed on the MED group only (Figure 6b) revealed that the first two principal components (PC1–PC2) explained 7.06% and 6.46% of the genetic variance, respectively. Most individuals clustered tightly around the origin. A subset of samples from the WGS_S2 dataset showed greater dispersion along PC1, extending towards positive values, while a few WGS_L samples were positioned higher along PC2. However, no distinct clustering patterns associated with the sequencing sets were observed.

The small clustering observed in the PCA (Figure 6b, bottom right) was consistent with the pedigree structure of the dataset. The average relatedness calculated using pedigree information (up to three generations) among the 24 individuals of the WGS_S2 dataset was very low, at 0.05 (s.d. of 0.2). However, the five animals forming the cluster showed a higher average relatedness among them, at 0.075 (s.d. of 0.06), whereas their relatedness with the remaining samples was only 0.002 (s.d. of 0.006). Pedigree inspection confirms that four of these five animals are paternal half-siblings, and the fifth originates from the same herd, which explains their greater genetic similarity. Moreover, they all belong to the same IBMI category, suggesting a possible influence of herd-level selection practices. Overall, the grouping highlighted in the PCA is, thus, primarily driven by pedigree structure and population stratification at the herd level, rather than recent inbreeding or analytical artifacts.

### 3.4. Annotation and Functional Variants

The annotated VCF file was explored to identify variants with a HIGH or MODERATE impact [48]. A total of 1217 variants (0.0087% of the total biallelic SNPs) in 1032 genes were classified as HIGH impact; in addition, 40,881 variants (0.29% of the total biallelic SNPs) in 11,354 genes were classified as missense variants. Among them, a subset of genes was considered because they were known, from the literature, to be associated with milk production and reproductive traits in buffalo (Table S4).

## 4. Discussion

The present work describes the creation of a variant database for buffalo, with a focus on the Mediterranean breed. A total of 41 million unique variants were detected in buffalo genomes. After the quality control process, over 11 million biallelic SNPs were classified as high-confidence biallelic SNPs polymorphic in the Mediterranean breed. This dataset provides a valuable resource for assessing the genetic variability of the breed and supports the development of genomic tools such as high-density SNP chips, widely used in genomic selection studies and precision breeding.

### 4.1. Limitations of Previous Genomic Tools

Earlier studies in buffalo have relied on SNP chips designed for the bovine genome due to the lack of a species-specific one, showing the generally low usefulness of the application of SNP chips across different species [49,50,51,52,53], or an SNP chip for buffalo, but designed using the bovine reference genome [24,54,55,56], due to the absence of a buffalo reference genome. In particular, the 90K SNP chip developed by Iamartino and colleagues [24], despite being based on the bovine genome (UMD3.1) as a reference, remains the most effective and cost-efficient tool currently available for genomic characterization of domestic buffalo [57].

The first draft reference genome for Mediterranean buffalo (UMD_CASPUR_WB_2.0) became available only in 2017 [58], providing the first resource for buffalo-specific analyses. Subsequent improvements on chromosome-level genome assembly (UOA_WB_1) [31] further enhanced buffalo-specific genomic research capabilities, providing more precise genetic mapping and analysis tools. Building on these advances, the most contiguous swamp buffalo genome assembly, PCC_UOA_SB_1v2 [59], achieved substantial resolution of telomeric and centromeric repeats and is approximately four-fold more contiguous than the existing river buffalo genome UOA_WB_1, even surpassing a recently published male swamp buffalo genome [60].

In the literature, examples of buffalo variant datasets are available. Iamartino and colleagues [24] identified approximately 22 million variants, currently not publicly accessible, through whole-genome sequencing of multiple buffalo breeds, including the Mediterranean buffalo. The scope was to create the Axiom Buffalo Genotyping 90K Array, designed to study genetic diversity and served as a starting point for genome-assisted selection programs in the Mediterranean [61]. A more recent example of a large genomic variant database (Buffgr) was provided by Khan and colleagues [62], who reported over 7.7 million SNPs, along with other genomic variants, identified across four buffalo breeds (Egyptian, Bangladesh, Jaffrarabadi, and Murrah) and mapped onto the same Mediterranean reference genome that we used. Also, this dataset is not currently publicly accessible. In this context, our dataset appears to be numerically rich in high-quality SNPs with low missingness (Figure 4) and higher variability (Figure 5), selected in the target population.

### 4.2. Methodological Innovation: Integration of Sequencing Technologies and Multi-Software Variant Calling

This study presents, for the first time in water buffalo research, a methodological innovation: the integration of two distinct sequencing technologies (long- and short-reads) for the purpose of calling genomic variants. This approach was employed to validate short-read sequencing results and provide additional confirmation [63]. Recent advances in long-read sequencing technologies have enabled more comprehensive approaches [64]. Although long-read sequencing is typically employed for detecting structural variants (SVs), Møller and colleagues (2020) [64] demonstrated that it can also resolve complex genomic regions often inaccessible to short-read sequencing, thereby improving the confidence of variant calling, including SNPs.

To further strengthen this strategy, variant calling was performed using multiple software tools [65]: GATK and GLnexus for long reads, maintaining only variants detected by both, and reducing the total number of SNPs but significantly increasing their accuracy; Bcftools and Freebayes for short reads [66,67]. The number of SNPs detected by the two short-read variant callers was strongly influenced by sequencing depth: WGS_S1 ranged from 3 to 40×, whereas WGS_S2 ranged from 28 to 56×. This feature appears to be relatively more important than the type of software used and the diversity of the samples. In fact, Cagirici and colleagues (2021) [68] showed that, when using the same aligner (BWA-MEM) on the same dataset, Bcftools achieves higher accuracy (i.e., a higher number of variants) than Freebayes. The lower depth of WGS_S1 likely explains its reduced variant resolution compared with WGS_S2, despite its broader breed composition. Overall, sequencing depth, one of the main factors influencing SNP calling accuracy and dataset completeness [69], was generally high across the datasets, with WGS_S1 as an exception. Consequently, dataset-specific genotype quality thresholds were applied during quality control. Some samples had very low coverage, as previously noted, and applying a stringent cutoff would have resulted in their exclusion. Therefore, the AAF per group was calculated using alternative allele counts rather than individual genotypes.

In addition, individuals and pools with insufficient coverage were excluded from downstream analyses requiring single-individual data, as they were not considered reliable.

### 4.3. Data Quality and Quality Control

In the WGS_S1 dataset, coverage was more variable than in WGS_S2 and WGS_L, which showed average values close to 99%. This variability was mainly due to the high number of samples with a sequencing depth below 10× and the minimum threshold for reliable variant calling [70], together with a high duplicate rate in pooled samples. Duplicates were marked and ignored during variant calling, as this procedure does not substantially affect SNP discovery [71]. Only one sample (SRR4477885) showed a high level of missingness and was excluded from downstream biodiversity analyses. In contrast, WGS_S2 and WGS_L samples had depths above 10×, resulting in higher and more uniform coverage.

Across all datasets, the average mapping rate exceeded 99%, with minor differences between WGS_S1 breed subsets (Mediterranean: 99.41%, river: 99.62%, and swamp: 99.07%), likely reflecting interspecies genetic divergence [59]. Overall, the high mapping rates indicated strong concordance with the reference genome and robust data quality [72].

### 4.4. Variant Calling Results

Overall, we detected 41 million unique variants and 14 million shared across all three datasets. An additional 18 million variants were identified exclusively in short-read data (i.e., with common variants only between WGS_S1 and WGS_S2). By integrating short- and long-read sequencing data and applying the proposed variant-calling strategy and quality control procedure, we ultimately generated a robust, high-confidence dataset of more than 11 million biallelic SNPs, characterized by high polymorphism (MAF > 0.02 in 99.9% of the initial dataset) and low missingness (CR > 0.9 in 78% of the initial dataset) within Mediterranean buffalo populations. Importantly, when considering only Mediterranean buffalo samples, the PCA (Figure 6b) showed no evidence of batch effects, suggesting that data integration across different sequencing datasets did not introduce technical biases. Furthermore, the variants identified were able to clearly separate the MED samples. A similar pattern is observed in PC3 of Figure S4, where MED samples are analyzed together with other buffalo populations. Comparison with other river buffalo breeds showed that 85.1% of SNPs were polymorphic, reflecting their shared evolutionary history, consistent with Presicce and colleagues [73] and Colli and colleagues [54]. PCA further supported this relationship, indicating a closer relationship between Mediterranean and river buffalo compared with swamp buffalo (Figure 6a). In contrast, the lower polymorphism in Swamp buffalo (43.8% of SNPs from the 11 million dataset) aligns with their independent domestication and substantial genetic divergence [74]. Similar results have already emerged in the study by Colli and colleagues [54] based on the 90k SNP chip, which has a moderate to high ascertainment bias: indeed, only 22.7% of array markers are polymorphic in swamp buffalo populations.

The distribution of variants across chromosomes was largely explained by chromosome size, confirming that most genomic variability increased proportionally with chromosome length (Figure 2) [75]. As expected, the largest autosomes (1–5) harbored the highest number of variants, whereas smaller chromosomes (21–24) showed markedly lower counts. The X chromosome contained fewer SNPs than autosomes of a similar size, while the Y chromosome and mitochondrial genome displayed the lowest numbers, consistent with their small size, conserved structure, and absence of recombination [76].

Across the entire genome, an average of 5624 SNPs per Mbp (±1680 SD) were identified. Although SNP density correlated with chromosome length, the proportion of unique and shared variants reflected both biological diversity and technical differences among the datasets. The high number of unique SNPs was mainly driven by the integration of different buffalo populations and by differences in sequencing depth, platforms, and variant-calling pipelines, which increase dataset-specific variant discovery.

Some chromosomal regions exhibited much higher SNP densities than others, highlighting areas that were either SNP-rich or SNP-poor. Among the most SNP-dense regions, in the one on chromosome 16 (35.00–36.00 Mbp), 71 genes were identified, all annotated as encoding olfactory receptors. Olfactory receptor genes are very common in mammals’ genomes (comprising ~5% of the genes) [77], and they accumulate a high number of variants due to the rapid evolution of these genes in response to environmental changes [78]. Another highly dense region was located on chromosome 13 (14.00–15.00Mbp), encompassing 11 genes in total, of which nine were uncharacterized genes and the DZIP1 and CLDN10 genes. The last two genes were already reported by Zwane and colleagues [79] in a similar region on chromosome 12 in cattle, which showed the top 1% overall SNP density. Chromosome 5 also displayed a peak in SNP density, between 66.00 and 67.00 Mbp, where only one uncharacterized gene was identified.

Among the SNP-poor regions, the window on chromosome 19 (57.00–58.00 Mbp) contained only three uncharacterized genes. On chromosome 11, a window (18.00–19.00 Mbp) included 23 genes, most of which were uncharacterized genes, together with PTGR2, a conserved gene involved in vasodilation and thermoregulation [80], and MIDEAS, an essential component of the MiDAC histone–deacetylase complex involved in chromosome alignment and embryonic development and a peroxisomal thioesterase [81]. Another SNP-poor window was detected on chromosome 8 (93.00–94.00 Mbp), which contains 16 genes; 12 are uncharacterized genes, and the others are PTGR2, NRF1, a nuclear transcription factor that controls mitochondrial gene expression and mtDNA maintenance [82], SMKR and UBE2H [83].

Despite the total number of variants obtained from variant calling being lower than that reported by Iamartino and colleagues [24], our dataset still provides a broad range of markers suitable for SNP chip selection. Indeed, considering a 90K SNP chip, which includes roughly three markers per 100 kbp window, or the BovineHD BeadChip, which includes 26 markers per window, our dataset could provide more than 460 markers per window, ensuring ample options for array design.

### 4.5. Candidate Genes for Productive and Reproductive Traits

Particular attention has been given to the identification of polymorphisms in buffalo proteins associated with production and reproduction characteristics (Tables S4 and S5):

1. Acetyl-CoA carboxylase alpha (*ACACA*): The *ACACA* enzyme catalyzes the first committed step of fatty acid synthesis in mammalian cytosol, the carboxylation of acetyl-CoA to malonyl-CoA, leading to the biosynthesis of long-chain fatty acids. In buffalo, therefore, it is an important candidate gene for the modulation of fatty acid composition in milk [84], as already observed in the bovine milk breed [85].
2. Casein alpha S1 (*CSN1S1*): The milk protein is essential for milk’s ability to transport calcium phosphate. Associations between the *CSN1S1* genotypes and milk production traits have also been proven in MED [86] and Murrah water buffalo [72].
3. Casein alpha S2 (*CSN1S2*): Casein alpha S2 is one of the phosphoproteins secreted in ruminants’ milk in the form of stable calcium–phosphate micelles, and it is the most hydrophilic of all caseins. A significant association between a non-synonymous SNP and the content of palmitic acid in buffalo milk has been observed [87].
4. Diacylglycerol acyl-CoA acyltransferase 1 (*DGAT1*): This enzyme catalyzes the final step in the formation of triglycerides, using diacylglycerol and acyl-CoA as substrates. Therefore, the *DGAT1* gene plays a pivotal role in milk and meat production of all ruminants [88]. In Murrah water buffalo, an association between *DGAT1* SNPs and milk production traits has been observed [89].
5. Melatonin receptor 1A (*MTNR1A*): This receptor is associated with seasonal reproductive activity in buffalo and milk protein percentage [90].
6. Fatty acid-binding proteins *FABP3* and *FABP4*: These proteins are members of the FABP multigene family. *FABP3* and *FABP4* have been found to be up-regulated during lactation and play a role in fatty acid trafficking towards milk triacylglycerol in buffalo [91].
7. Thyrotropin-releasing hormone-degrading enzyme (*TRHDE*): This enzyme is an extracellular enzyme that breaks down the thyrotropin-releasing hormone (TRH). Lactotrope-specific downregulation of *TRHDE* was associated with high milk production in river buffalo [92].
8. Oxytocin/neurophysin I prepropeptide (*OXT*): Oxytocin is a hormone produced in the hypothalamus. It is a candidate gene for improving milk yield and milkability in MED due to the role of the oxytocin hormone in alveolar milk ejection and in milk flow rate [93].
9. Thyroglobulin (*TG*): TG is a glycoprotein hormone, synthesized in thyroid follicular cells, and is a carrier for both triiodothyronine (T3) and thyroxine (T4), stored in the thyroid gland. An association between SNPs in the *TG* promoter region and milk production traits has been reported in river buffalo [94].
10. Oxytocin receptor (*OXTR*): The oxytocin–oxytocin receptor complex plays an important role in the uterus during calving, and one of the two variants identified by us has been associated with milk fatty acid composition in MED [95].
11. Sterol regulatory element-binding protein (*SREBP*) cleavage-activating protein (SCAP): This protein is a key regulator of cholesterol homeostasis in cells. Missense SCAP SNPs have been associated with milk production traits in water buffaloes [96].
12. Stearoyl-CoA desaturase (*SCD*): This endoplasmic reticulum enzyme plays an essential role in cellular biosynthesis of monounsaturated fatty acids, and it is involved in the endogenous production of the cis-9, trans-11 isomers of CLA. A SNP in the promoter region of the *SCD* gene has been associated with daily milk yield and milk fat traits in Italian river buffalo [97].

Most of the related coding genes have already been found to be associated with these characters in ruminants, but not always in MED. The roles of all the missense variants identified by us have not been exhaustively investigated in MED, suggesting that our dataset could potentially play a role in boosting research in the field.

The variant analysis revealed notable allelic diversity across genes involved in milk production, lipid metabolism, immune response, and reproductive regulation.

Among the genes involved in milk synthesis and composition, variants were identified in *ACACA*, *FABP3*, *DGAT1*, *CSN1S1*, *CSN1S2*, and *TG* genes that exhibited population-specific frequency differences, suggesting potential impacts on fatty acid synthesis, protein composition, and overall milk quality. For example, several missense variants in DGAT1 and TG showed high frequencies in all breeds.

Regulatory genes of lactation and growth, such as the *PRL* [98], *PRLR* [99], *OXT* [93], *MTNR1A, TRHDE, SCAP*, and *GHRL* [100] genes, also displayed high allelic patterns in the river group compared with the swamp or Mediterranean groups. Variants in the *ABCG2* [101] and *PPARGC1A* [102] genes were identified, and they were mostly present in Mediterranean samples. The two genes have a role in lipid metabolism modulation and nutrient transport.

The clearest differentiation, however, appeared in genes involved in innate immunity, *TLR2* [103], *TLR4* [104], *MBL2* [105], *C3* [56], and *TNF* genes. Swamp populations exhibited greater allelic diversity, and river populations showed intermediate profiles, whereas Mediterranean buffalo appeared more homogeneous, with AAF mean (and SD) values of 0.511 ± 0.329, 0.437 ± 0.254, and 0.448 ± 0.179, respectively. Genes involved in hormonal regulation and nutrient transport, such as *OXT, OXTR* [95], *FCGRT* [106], and *LTF* [107], had average (and SD) AAF values of 0.271 ± 0.261, 0.369 ± 0.303 and 0.331 ± 0.151 in swamp, river and Mediterranean buffalo.

Regarding these genes, we identified SNPs that have been previously reported in the literature. In *CSN1S2*, the SNP A > G in exon 16, which causes a threonine (Thr)-to-alanine (Ala) substitution at position 190, was significantly associated with palmitic acid content in buffalo milk [87]. In *PRL*, the T > C polymorphism in exon 2, resulting in an arginine (Arg)-to-cysteine (Cys) substitution at position 12, showed a notable effect on milk fat content [98]. In *OXT*, the G > T SNP in exon 2 leads to an arginine (Arg)-to-leucine (Leu) substitution at position 97 [93]. Finally, in *OXTR,* the C > T SNP in exon 3 causes an arginine (Arg)-to-cysteine (Cys) substitution at position 353 and was associated with significant effects on milk fatty acid composition traits [95].

### 4.6. Inclusion of the Y Chromosome

Another insight of this study is the integration of the Y chromosome. While autosomal and X chromosome variation has been extensively characterized, the Y chromosome has remained largely unexplored in water buffalo genomics. Only in 2022, a first draft of the Y chromosome in Swamp buffalo was published [60], but none for the Mediterranean breed. To incorporate biallelic Y-linked SNPs into our database, we included the Y chromosome of the Yak (*B. grunniens*) from the BosGru3.1 assembly (2021) to the UOA_WB_1 (2019) buffalo reference. The Yak Y chromosome was chosen for its close phylogenetic relationship with water buffalo [108] and the superior assembly quality of its genome compared with other bovids [109].

Additional technical constraints contributed to the reduced representation of the Y chromosome in our study: the WGS_S2 dataset consisted exclusively of females, and long-read sequencing data showed poor and uneven alignment on the Y chromosome due to its high repeat content, half sequencing depth due to its haploid nature, and high similarity to some regions of the X chromosome [60]. As a result, Y-linked variants could only be retrieved from the WGS_S1 dataset, inevitably limiting the breadth of detectable polymorphisms.

Despite these challenges, we identified 1350 biallelic SNPs on the Y chromosome, corresponding to a mean density of ~50 SNPs/Mbp (±152.49 SD). These numbers reflect the notoriously complex nature of the Y chromosome, whose X-degenerate genes and repetitive blocks challenge even the most advanced sequencing technologies [76]. Despite low variant numbers, Y-specific markers remain valuable for reconstructing paternal haplotypes and potentially improving marker-assisted selection [110] also in reproductive traits [76,111]. However, at this moment, this analysis is limited by the absence of a Mediterranean buffalo Y chromosome reference; further analyses need to be done. For these reasons, the integration of Y-linked variants into our database and their consideration in the design of the future SNP array are justified and add biological, evolutionary and practical value.

### 4.7. Implications for Mediterranean Buffalo Breeding

Current genomic investigations in buffalo, especially in the Mediterranean breed, have largely relied on bovine-derived SNP arrays due to the gap in breed-specific genomic resources. The high-confidence biallelic SNP catalog presented here could overcome this limitation by incorporating millions of variants identified directly from this species, including also Y chromosome markers derived from the closely related Yak reference sequence. Thus, this unbiased and population-tailored variant set captures the true genetic variability of the target breed, enabling a rapid development of molecular diagnostics and functional assays, or used to go deeper from a functional point of view for GWAS and evolutionary studies without any additional experimental effort. Consequently, the development of tailored tools that better capture population-specific variability than standard ones has the potential to improve the accuracy of genomic studies in buffalo breeding programs, as demonstrated for bovine species [112,113].

## 5. Conclusions

The SNP dataset here identified constitutes the most densely characterized and breed-specific genomic variant catalog currently available for Mediterranean buffalo. This resource not only strengthens the genomic foundation for studies within this breed but also provides a valuable reference for comparative analyses across other buffalo populations, such as river and swamp. By comparing the SNP effects on economically and biologically relevant phenotypes, this study supports broader breed strategies and market-driven improvements within the buffalo production sector.

## Data Availability

The data presented in this study are available upon request from the corresponding author due to the data being part of an ongoing study.

## Supplementary Materials

The following supporting information can be downloaded at https://www.mdpi.com/article/10.3390/ani16060922/s1. Figure S1: Distribution of genetic variant types across buffalo chromosomes. The stacked bar plot shows the number of identified variants per chromosome, classified as bi-allelic single nucleotide polymorphisms (Bi-allelic SNP) in blue, multi-allelic SNPs (Multi-allelic SNP) in violet, insertions/deletions (Bi-allelic INDEL) in green and multi-allelic insertions/deletions (Multi-allelic INDEL) in orange. Chromosomes are arranged sequentially from 1 to 24, including the X, Y, and mitochondrial (MT) chromosomes; Figure S2: Venn diagram. The diagram illustrates the overlap of bi-allelic SNPs among the three genomic datasets: WGS_S1, WGS_S2 and WGS_L. Numbers in each section represent the count of SNPs unique to each dataset or shared between two or all three datasets. The intersection set, common to all datasets, was used for downstream analyses; Figure S3: Hierarchical clustering dendrogram. The dendrogram shows the genetic relationships among populations. The branch lengths represent genetic distances, indicating how closely related the populations are. Colors denote the Mediterranean (green), River (blue), and Swamp populations (yellow); Figure S4: Principal Component Analysis (PCA) based on SNP genotypes (PC1 vs PC3). Each point represents an individual: green indicates the Mediterranean population, blue the other River populations, and yellow the Swamp population. The relative distances among points reflect the genetic similarity or divergence among individuals; Table S1: List of samples. The samples were downloaded from NCBI SRA, categorized by subspecies, breed, and accession ID [114,115]; Table S2: Summary of alignment metrics for the analysed samples. The total number of raw (Reads raw) and mapped reads (Mapped reads), the proportion of successfully aligned reads (Percent reads mapped), the number of properly paired (Properly paired reads) and duplicate reads (Duplicates), and the mean sequencing depth (Depth) are reported. The NA value for the long-read dataset (WGS_L) is due to differences in the alignment metrics generated by the technology, which does not account for properly paired reads or duplicates; Table S3: Density and distribution of genetic markers across chromosomes. The table shows, for each chromosome (Chr), the total number of genotyped markers (N_markers), chromosome length in base pairs (Length_bp), marker density per megabase (Density_Mb), and average distance between markers (Distance_avg). Chromosomes include autosomes (1–24), sex chromosomes (X, Y), and the mitochondrial chromosome (MT); Table S4: Genes associated with reproductive and milk production traits reported in the literature. For each gene, its name, relevant reference are provided and the number of observed variants, regardless of the variant type classification; Table S5: Summary of identified variants. The table lists, for each variant, the chromosome (Chr), genomic position (Pos), observed frequencies in different groups or populations (Freq MED, Freq RIV, Freq SWA), reference (REF) and alternative (ALT) alleles, predicted functional impact (Impact), variant type (Type of variant), affected gene (Gene), amino acid change in the correctly assembled trascript (aminoacid). The green cells identify the SNPs known in literature.

## Abbreviations

The following abbreviations are used in this manuscript:

AAF	Alternative allele frequency
AD	Allele depth
ANASB	Associazione Nazionale Allevatori Specie Bufalina
CR	Call rate
DP	Total depth
ENA	European Nucleotide Archive
InDels	Insertions/deletions
MAF	Minor allele frequency
MED	Mediterranean
MNPs	Multi-nucleotide polymorphisms
ONT	Oxford Nanopore Technologies
PCA	Principal component analysis
PDO	Protected designation of origin
RIV	River
WGS	Whole-genome sequencing
