# Supplementary material for: Exploring Genomic Variability in the Mediterranean Buffalo Breed: A Step Towards Custom SNP Array
Source: Animals (Basel). 2026 Mar 15;16(6):922. doi: 10.3390/ani16060922 (PMC13023267; doi:10.3390/ani16060922)
Supplement: Supplementary file 1 [file animals-16-00922-s001.zip › Supplementary_rev04_13032026/Supplementary materials.docx]

**Supplementary materials**


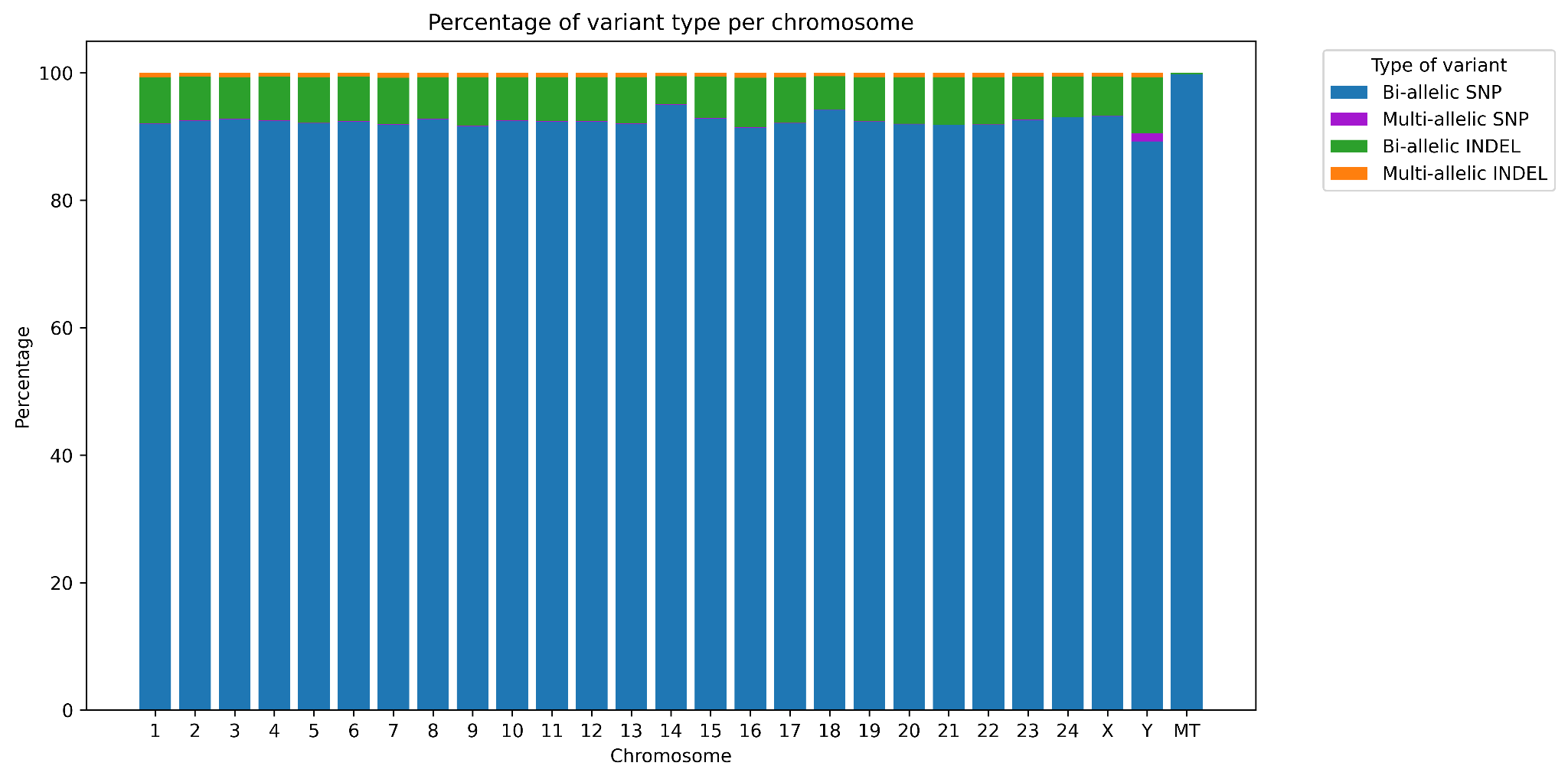


Figure S1: Distribution of genetic variant types across buffalo chromosomes. The stacked bar plot shows the number of identified variants per chromosome, classified as bi-allelic single nucleotide polymorphisms (Bi-allelic SNP) in blue, multi-allelic SNPs (Multi-allelic SNP) in violet, insertions/deletions (Bi-allelic INDEL) in green and multi-allelic insertions/deletions (Multi-allelic INDEL) in orange. Chromosomes are arranged sequentially from 1 to 24, including the X, Y, and mitochondrial (MT) chromosomes.


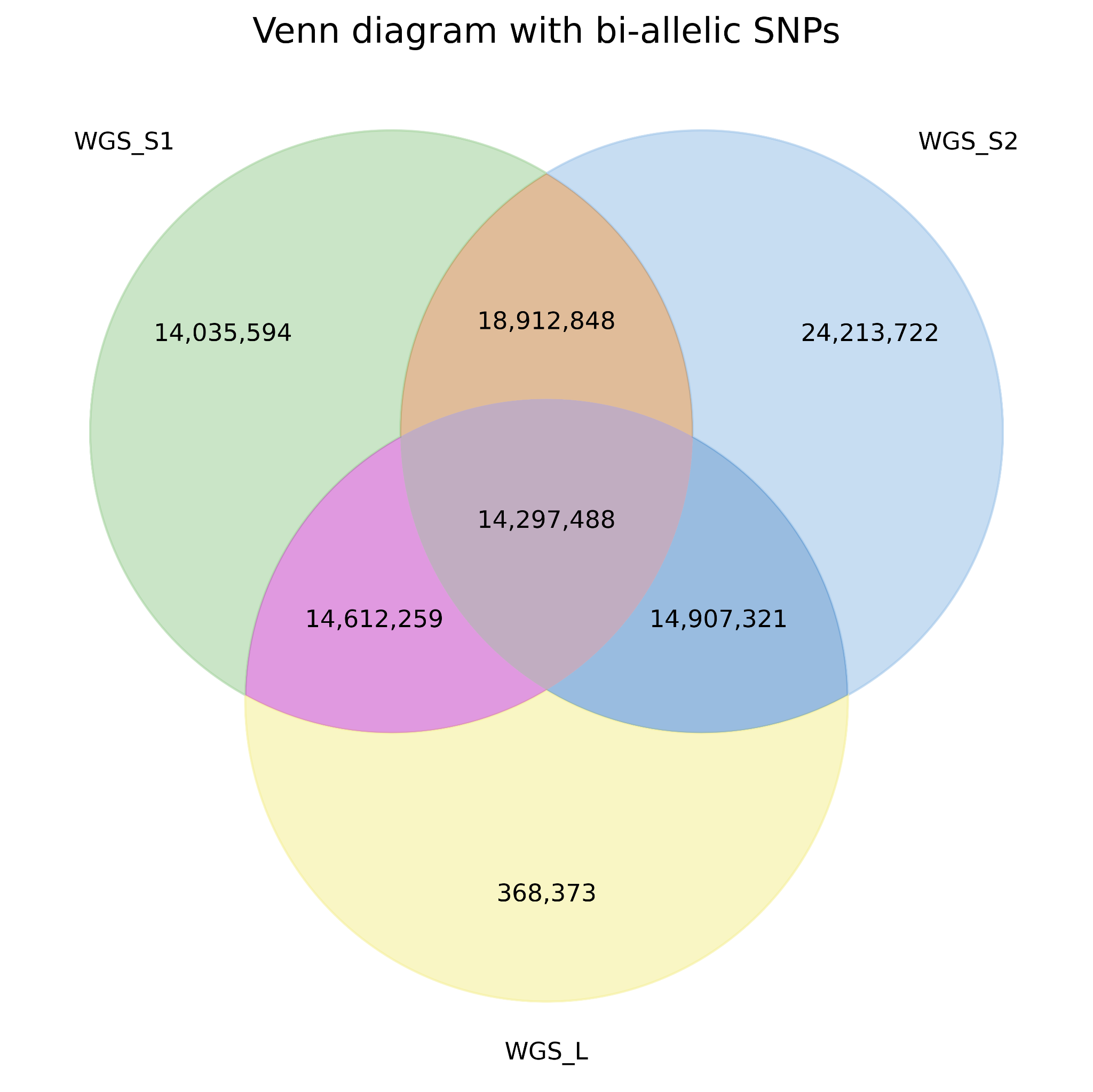


Figure S2: Venn diagram. The diagram illustrates the overlap of bi-allelic SNPs among the three genomic datasets: WGS_S1, WGS_S2 and WGS_L. Numbers in each section represent the count of SNPs unique to each dataset or shared between two or all three datasets. The intersection set, common to all datasets, was used for downstream analyses.


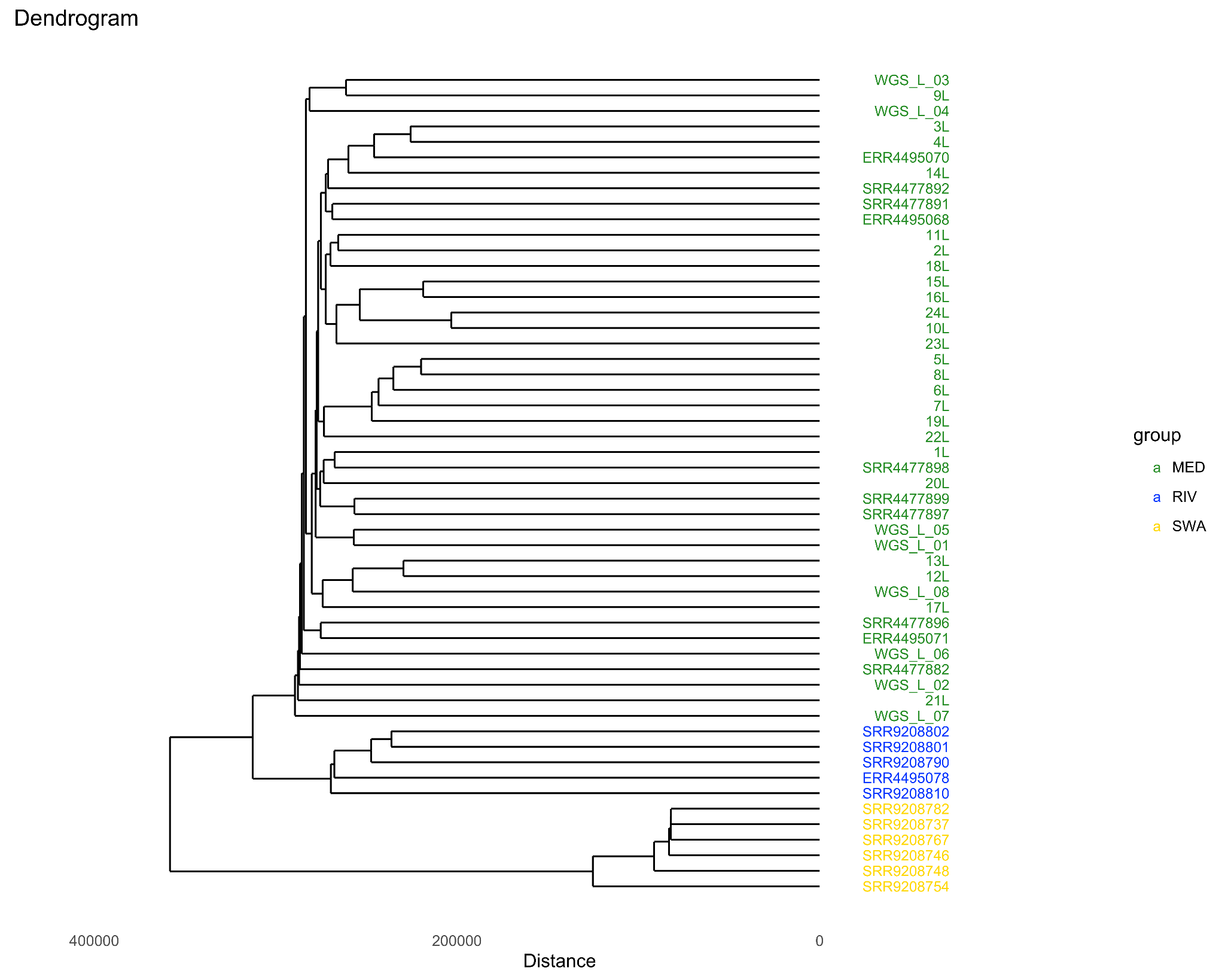


Figure S3: Hierarchical clustering dendrogram. The dendrogram shows the genetic relationships among populations. The branch lengths represent genetic distances, indicating how closely related the populations are. Colors denote the Mediterranean (green), River (blue), and Swamp populations (yellow).


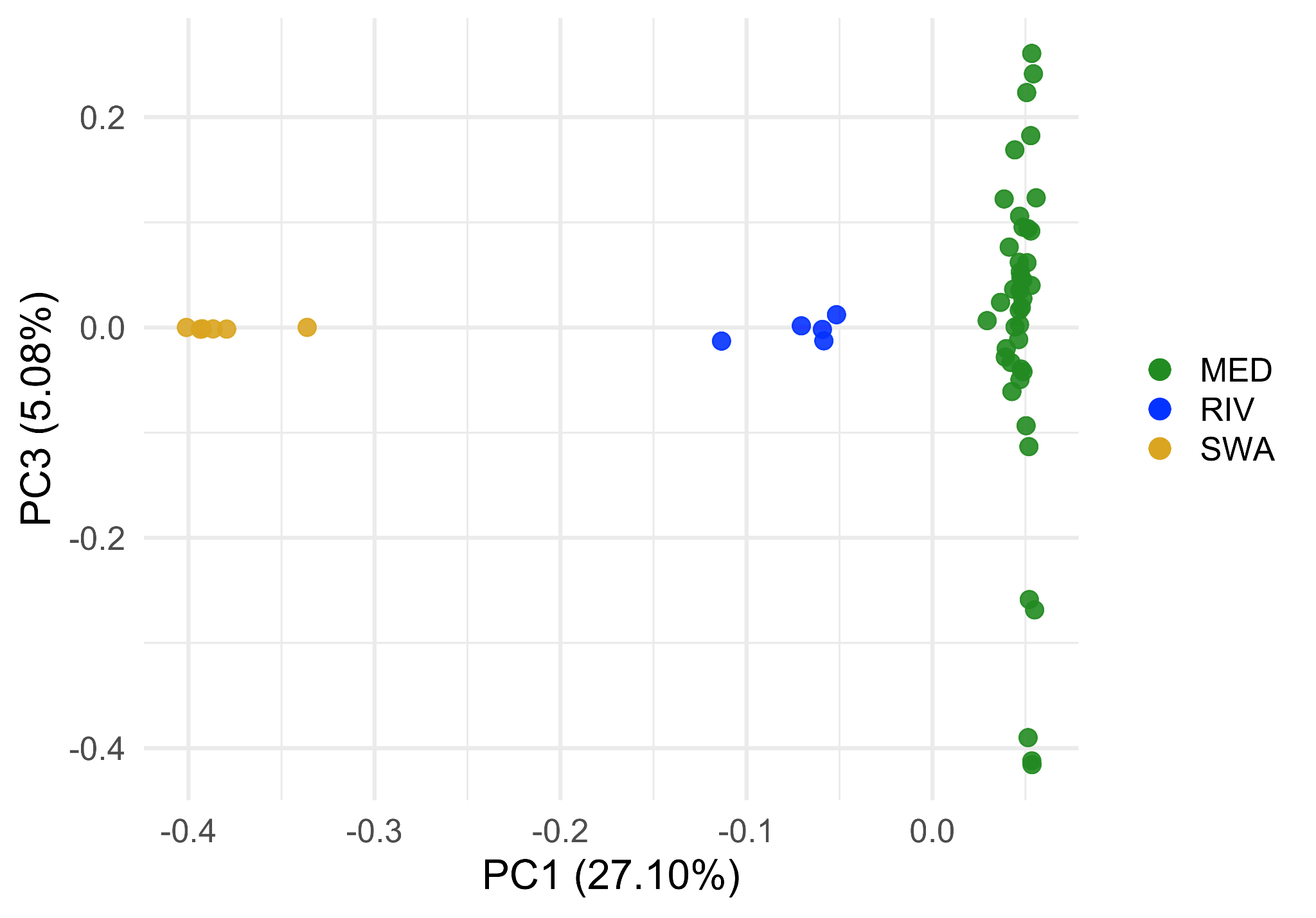


Figure S4: Principal Component Analysis (PCA) based on SNP genotypes (PC1 vs PC3). Each point represents an individual: green indicates the Mediterranean population, blue the other River populations, and yellow the Swamp population. The relative distances among points reflect the genetic similarity or divergence among individuals.
