# Supplementary figures and images for: Exploring Genomic Variability in the Mediterranean Buffalo Breed: A Step Towards Custom SNP Array
Source: Animals (Basel). 2026 Mar 15;16(6):922. doi: 10.3390/ani16060922 (PMC13023267; doi:10.3390/ani16060922)

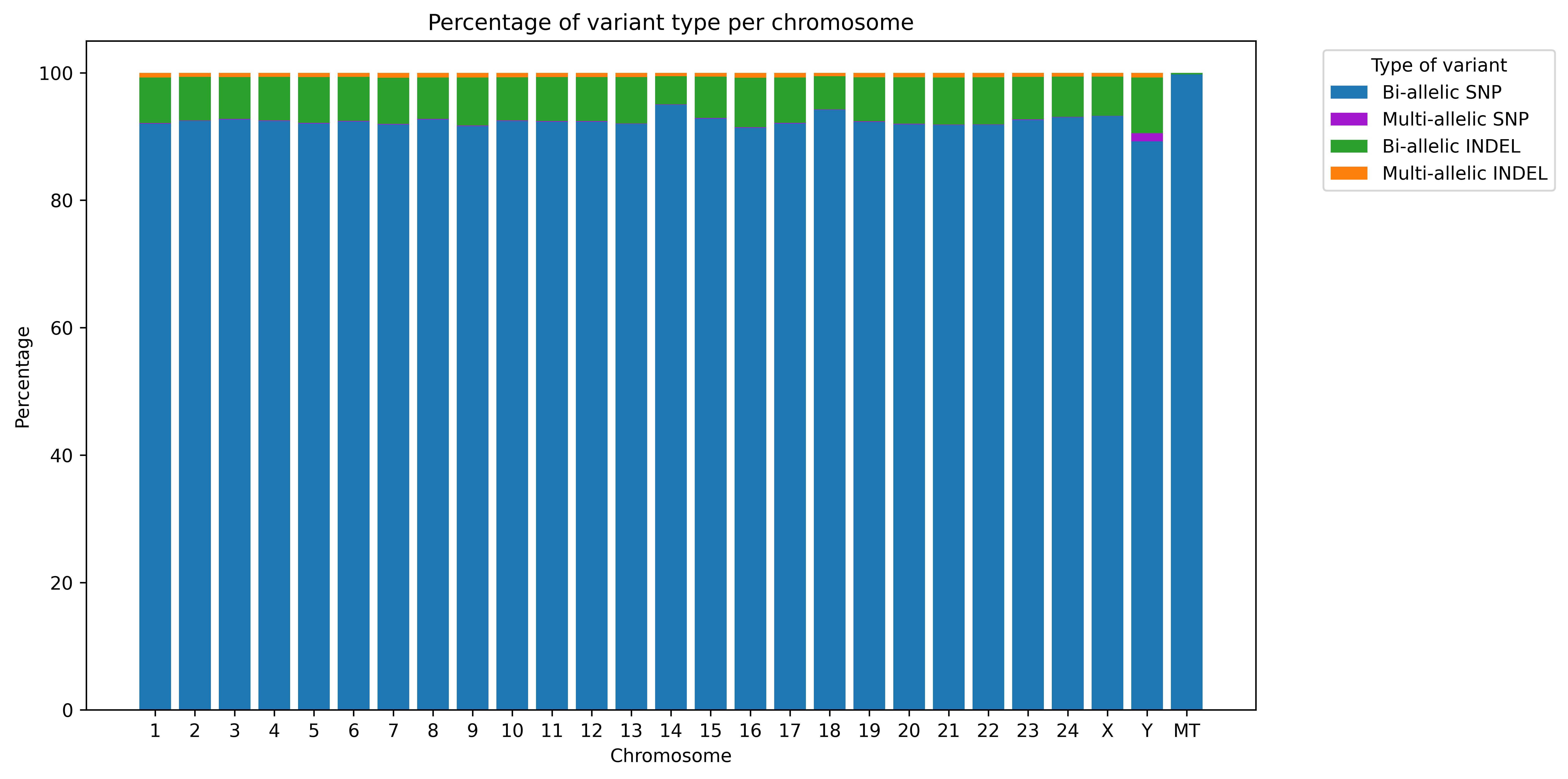

Supplement: Supplementary file 1 [file animals-16-00922-s001.zip › Supplementary_rev04_13032026/S1.png]

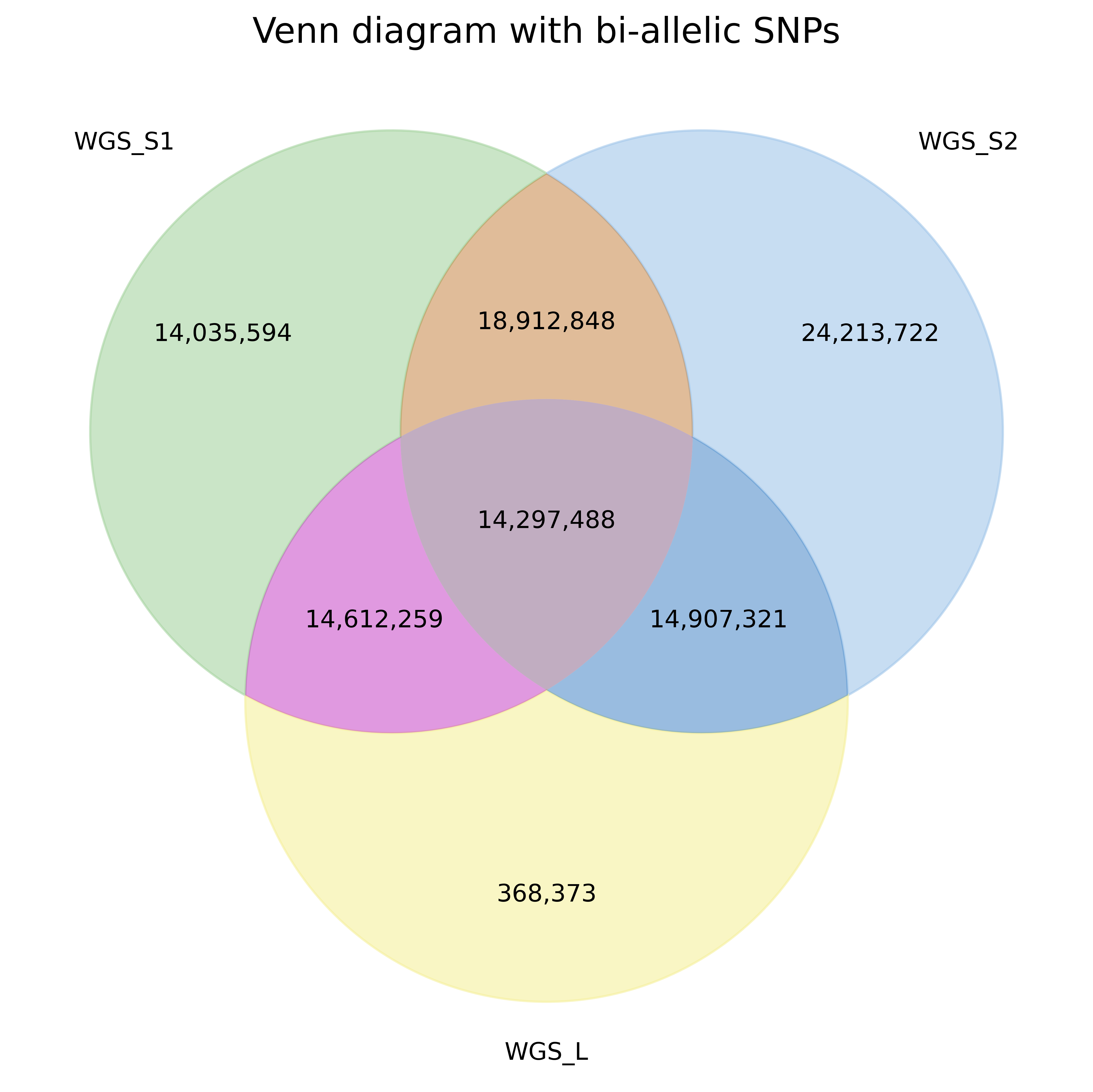

Supplement: Supplementary file 1 [file animals-16-00922-s001.zip › Supplementary_rev04_13032026/S2.png]

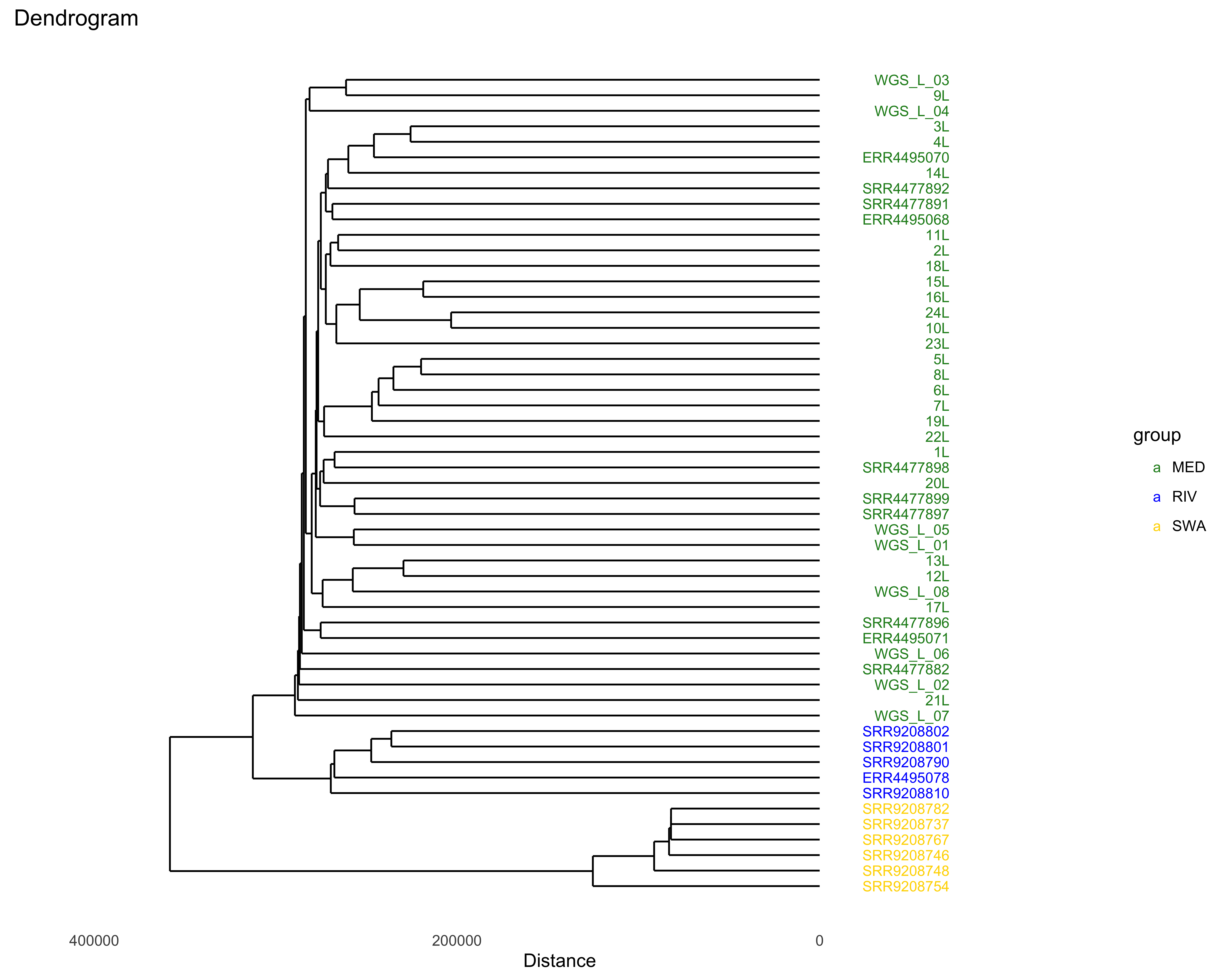

Supplement: Supplementary file 1 [file animals-16-00922-s001.zip › Supplementary_rev04_13032026/S3.png]

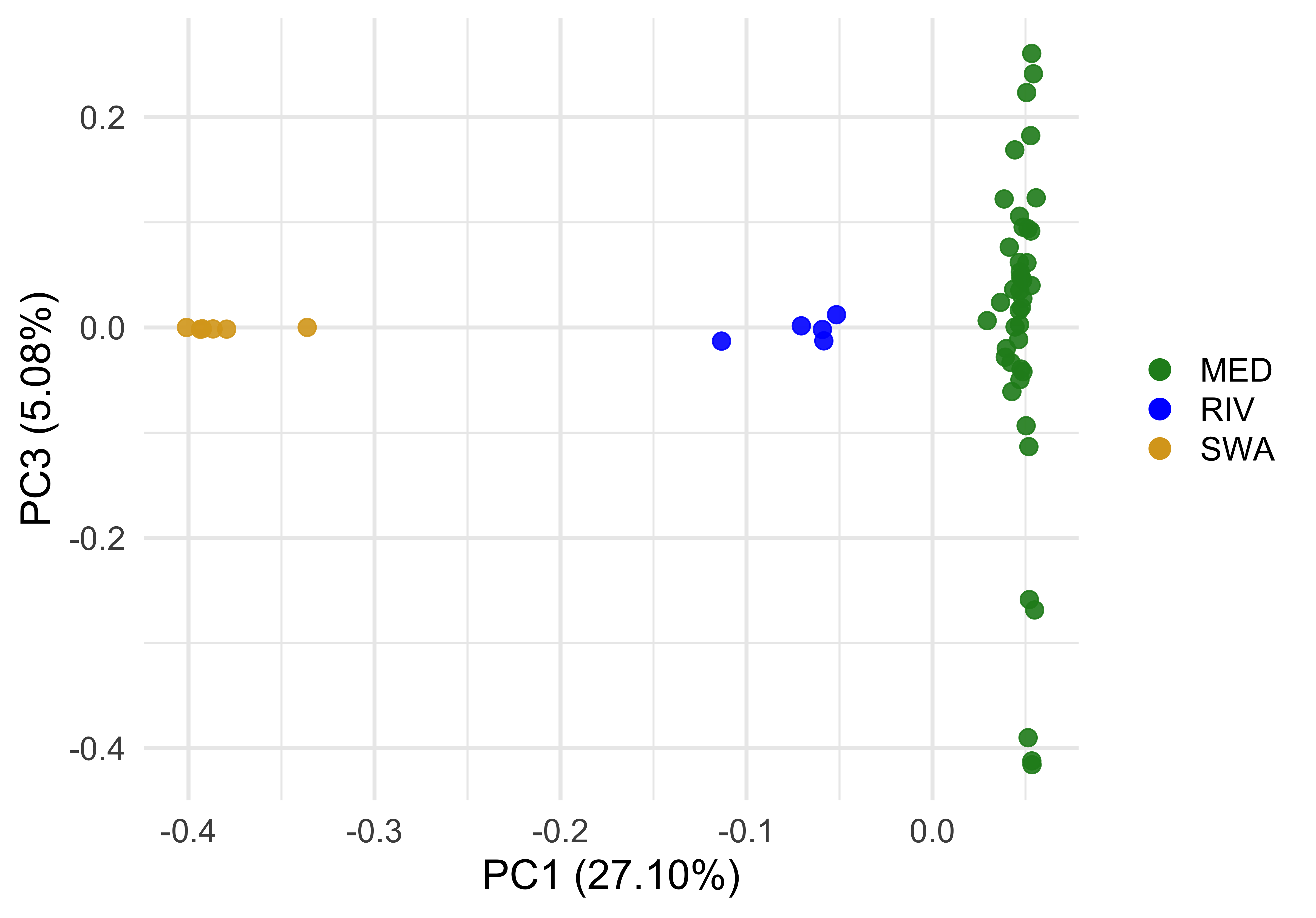

Supplement: Supplementary file 1 [file animals-16-00922-s001.zip › Supplementary_rev04_13032026/S4.png]
